# A Bias-Corrected HighResMIP Dataset for Impact Assessment Studies

**DOI:** 10.1038/s41597-026-07709-y

**Published:** 2026-07-02

**Authors:** Fuseini Yakubu, Jürgen Böhner, Laurens M. Bouwer, Shabeh ul Hasson

**Affiliations:** 1 HAREME Lab, Institute of Geography, Earth and Society Research Hub, Universität Hamburg, Hamburg, Germany; 2 Climate Service Center Germany (GERICS), Helmholtz-Zentrum Hereon, Hamburg, Germany; 3 Institute of Geography, Earth and Society Research Hub, Universität Hamburg, Hamburg, Germany

## Abstract

Climate impact assessments increasingly require high-resolution climate projections that capture fine-scale processes. While the High Resolution Model Intercomparison Project (HighResMIP) provides global climate simulations at 25–50 km resolution without statistical downscaling, systematic biases still limit their direct application for impact assessment studies. Here we present BC-HiRMIP, the first comprehensive globally bias-adjusted HighResMIP experiments at daily temporal and 0.5° spatial resolution, covering the period 1979-2050. Across four global climate models (MPI-ESM1-2-XR, EC-Earth3P-HR, CNRM-CM6-1-HR, HadGEM3-GC31-HM), BC-HiRMIP includes up to 11 essential meteorological variables (temperature, precipitation, humidity, radiation, wind, and pressure), spanning equilibrium climate sensitivities of 2.99-5.62 °C. The datasets were bias adjusted using the ISIMIP3BASD v3.0.1 methodology that preserves model-projected climate change signals across distribution quantiles with W5E5 v2.0 as reference. Comprehensive validation across diverse climate zones demonstrates substantial bias reduction, minor differences between raw and bias-adjusted climate change signals and distributional characteristics. This standardized, multi-variable, multi-model dataset bridges the gap between climate modeling capabilities and impact assessment needs, enabling applications in hydrology, agriculture, renewable energy, and climate service research.

## Background & Summary

Climate impact assessments increasingly require high-resolution climate projections that capture fine-scale processes such as orographic precipitation, coastal gradients, extreme events, and terrain-modified temperature patterns^1^. The Coupled Model Intercomparison Project Phase 6^2^ (CMIP6) has provided a firm foundation for climate science and services, yet most CMIP6 models still operate at ~100 km horizontal resolution and above, making them too coarse for many regional and local applications^2,3^. To improve the resolution of these coarse Earth System Models (ESMs) and enhance its use at the local scale, two approaches have dominated literature: statistical downscaling and dynamical downscaling using Regional Climate Models (RCMs)^4^. Statistical downscaling uses statistical relationships between large-scale climate variables (from global climate models) and local observations to predict future local conditions, assuming these relationships remain stable over time while dynamical downscaling embeds high-resolution regional climate models within coarser global climate models to simulate local climate processes through physical equations, capturing regional dynamics like topography and land-sea contrasts^5^. However, both approaches introduce additional uncertainties and processing chains that sometimes, compound the inherent model biases^6^. Statistical downscaling assumes stationarity in scale relationships that may not hold under climate change, while RCM simulations remain computationally expensive, and typically covering limited domains rather than the entire globe^7^.

The High Resolution Model Intercomparison Project^3^ (HighResMIP) was established within CMIP6 to explore the benefits of increased resolution in global climate models, with participating models reaching 25–50 km horizontal resolution, comparable to many regional models but with global coverage^8,9^. Studies using HighResMIP experiments have demonstrated improved representation of extreme temperatures, intense precipitation events, and regional climate features compared to standard-resolution CMIP6 models, with particularly notable gains in regions of complex topography and coastal zones^8–10^. The improved representation of topographic features and coastal gradients at HighResMIP resolution is particularly valuable for impact assessments in mountainous regions, small island states, and coastal zones where coarser models systematically underestimate local climate variability and extreme event intensities^11^. Over 160 peer-reviewed studies have utilized HighResMIP experiments across diverse applications including extreme events analysis, monsoon dynamics, tropical cyclone projections, and ocean-atmosphere interactions according to the HighResMIP publication list (https://highresmip.org/about/publications/, Last access: 08 December 2025). HighResMIP experiments provide historic simulations (1950–2014) and near-term projections (2015–2050) under high-emission scenarios referred to as highres-future. While this temporal scope differs from the century-scale projections typical of standard CMIP6 (extending to 2100), the 1950-2050 window aligns well with near-term impact assessment needs, providing data relevant for immediate adaptation planning such as decisions for infrastructure, urban planning, water resources, and agricultural systems.

For example, hydrological models used for water resource planning are highly sensitive to precipitation phase partitioning (rain versus snow), elevation-dependent temperature gradients, and the spatial distribution of precipitation, all areas where HighResMIP’s native resolution offers potential advantages^12,13^. Recent studies show that hydrological projections, especially for extreme flows and snowmelt-driven floods, exhibit substantial sensitivity to both climate forcing resolution and bias adjustment quality^13,14^. Agricultural modeling requires comprehensive meteorological forcing including temperature extremes, precipitation, radiation, and humidity. Cryosphere applications, including snow hydrology, permafrost dynamics, and glacier modeling, are particularly sensitive to precipitation phase and elevation-dependent gradients, and here HighResMIP’s high resolution offers potential advantages^12,13,15^. The renewable energy sector also requires accurate wind and solar radiation data, where HighResMIP’s improved representation of topographic channelling and cloud processes could enhance resource assessments if biases are well addressed^16^.

However, like all climate models, HighResMIP simulations also exhibit systematic biases when compared to observations, particularly in precipitation intensity, temperature extremes, and regional climate patterns^9,17,18^. These biases limit the direct application of HighResMIP outputs in impact assessments, without statistical bias adjustments. Despite the widespread availability of bias-adjusted CMIP6 products (e.g. ISIMIP3b^19^, NEX-GDDP-CMIP6^20^), no comprehensive bias-adjusted HighResMIP experiments have been made publicly available yet. Whereas ISIMIP3b^19^ and NEX-GDDP-CMIP6^20^ provide bias-adjusted and statistically downscaled CMIP6 products at relative finer resolutions, researchers have to choose between accepting resolution limitations (coarser GCMs) or performing computationally intensive custom bias adjustments, a significant barrier noted in multiple recent impact studies^20^.

Beyond specific application domains, the climate services community has identified a broader need for standardized, well-documented and high quality climate datasets that enable reproducible research and operational applications. Frameworks such as the ISIMIP3^19^ and NEX-GDDP-CMIP6^20^ have successfully established such standards for CMIP5 and CMIP6 data, creating a common reference that facilitates multi-model impact assessments and cross-study comparisons. However, HighResMIP is yet to be fully integrated into such a standardized framework despite its potential added value. The lack of standardized HighResMIP products thus represents a significant gap between the climate modeling community’s technical capabilities and the practical needs of the impact assessment and the adaptation planning communities.

Here we present a global bias-adjusted HighResMIP dataset that, to our knowledge, constitutes the first publicly available multi-model, multi-variable HighResMIP product prepared following a standardized framework. This dataset covers 11 essential meteorological variables from four HighResMIP experiments referred to as Bias-Corrected HighResMIP Dataset for Impact Assessment Studies^21^ (BC-HiRMIP). The BC-HiRMIP^21^ dataset spans 1979–2050, covering both historical simulations (1979–2014) and the high-emission future scenario (highres-future, 2015–2050). BC-HiRMIP^21^ uniquely provides bias-adjusted data from native high-resolution global models (25–50 km), which are horizontally remapped to 0.5° but do not undergo any additional statistical downscaling step beyond the bias adjustment. This approach reduces compounding uncertainties associated with further statistical downscaling, filling a critical gap for applications requiring physically consistent high-resolution forcing that captures fine-scale processes with less uncertainty^6,7,22,23^. This gap is particularly critical given the growing demand for high-resolution climate data in applications such as Adaptation, Climate Services, Vulnerability and Impact Assessment^22,24,25^.

We selected variables following established impact modeling frameworks, including temperature variables (mean (tas), minimum (tasmin), maximum (tasmax)), precipitation (total (pr) and snowfall (prsn)), relative (hurs) and specific humidity (huss), surface pressure (ps), wind speed (sfcWind), and shortwave (rsds) and longwave (rlds) radiation. All variables except tasmax, tasmin, prsn and huss were bias-adjusted directly using the ISIMIP3BASD method^26^ with the W5E5 v2.0 dataset^27^ as the reference. These exempted variables were derived using indirect approaches after bias adjustment of the other variables. The four models used in forming BC-HiRMIP^21^ include, MPI-ESM1-2-XR^28^, EC-Earth3P-HR^29^, CNRM-CM6-1-HR^30^, and HadGEM3-GC31-HM^31^, herein referred to as MPI-XR, EC-Earth3P, CNRM, and HadGEM3, respectively (see Technical Validation and Table 1 for detailed summary). These experiment also span a wide range of equilibrium climate sensitivities (ECS), ranging from 2.99 to 5.62 °C, while also representing diverse modeling centers and model parameterisations^32^.

**Table 1:** List of HighResMIP experiments bias-adjusted to form the BC-HiRMIP dataset.

ID	HighResMIP	Variant Label	Resolution	ECS (K)	Group	Country
1.	MPI-ESM1-2-XR	r1i1p1f1	0.47°	2.99	Max Planck Institute for Meteorology	Germany
2.	EC-Earth3P-HR	r1i1p2f1	0.35°	4.22	EC-Earth-Consortium, Rossby Center, Swedish Meteorological and Hydrological Institute/SMHI	Sweden
3.	CNRM-CM6-1-HR	r1i1p1f2	0.5°	4.94	Centre National de Recherches Meteorologiques, Centre Europeen de Recherche et de Formation Avancee en Calcul Scientifique	France
4.	HadGEM3-GC31-HM	r1i1p1f1	0.35°	5.62	Met Office Hadley Centre	United Kingdom

## Methods

### Reference climate dataset

In this study, the W5E5 v2.0^27,33^ from the ISIMIP repository (10.48364/ISIMIP.342217) was used as a reference input for bias adjusting the HighResMIP experiments. W5E5 v2.0^27,33^ is a global merged dataset that combined WFDE5^33,34^ data over land areas and ERA5^35^ data over the oceans into one. Over both land and ocean, precipitation is bias-adjusted against monthly totals from the Global Precipitation Climatology Project v2.3^36^ (GPCP), ensuring consistency with observational precipitation estimates. It is worth noting that, W5E5 v2.0^27,33^ is largely based on model products. W5E5 v2.0^27,33^ was used for bias adjusting datasets in the ISIMIP3b^19^, providing a global coverage for several impact assessment variables. It has a daily temporal resolution and a horizontal spatial resolution of 0.5°, providing a consistent global coverage from 1979-2019. This study uses 1979-2014 as the overlapping period with the period 1950-2014 from the HighResMIP experiments.

### HighResMIP Experiments

Four models partaking in the HighResMIP experiments were selected. These models includes MPI-ESM1-2-XR^28^, EC-Earth3P-HR^29^, CNRM-CM6-1-HR^30^, and HadGEM3-GC31-HM^31^. These four models represent all HighResMIP participants providing complete coverage of both the historic period (1950–2014) and future projections (2015-2050) with the required suite of impact-relevant variables at the time of preparing this dataset. All HighResMIP experiments used in this study are freely available from the Earth System Grid Federation (ESGF) Archive (https://esgf-metagrid.cloud.dkrz.de, Last access: 8 December 2025). The highres-future simulations follow the SSP5-8.5 scenario, designed to approximate CMIP5 RCP8.5 forcing trajectories while incorporating the updated socioeconomic Pathway framework^9,37^. This provides coverage for several relevant climate variables for the impact modelling community including tas, tasmin, tasmax, pr, prsn, hurs, huss, rlds, rsds, sfcWind and ps. These experiments provide a wide range of ECS 2.99-5.62 °C, providing the impact assessment community the opportunity to quantify different model responses to various warming trajectories^24,32^. Table 1 provides detailed information on each of the experiments used to form the BC-HiRMIP^21^ dataset.

### Preprocessing dataset

For HadGEM3, which uses a 360-day calendar (12 months of 30 days each), we converted the data to a standard Gregorian calendar because the bias-adjustment framework requires calendar alignment based on Gregorian days and months. While the ISIMIP3b^38^ fact sheet recommends harmonization to a proleptic Gregorian calendar, it does not prescribe a specific implementation. Therefore, we adopted the following approach: (i) removing one or two ‘pseudo’ days from February (two days for non-leap years and one day for leap years), and (ii) linearly interpolating values to generate the missing 31st day in Gregorian months with 31 days (January, March, May, July, August, October, and December), which are absent in the 360-day calendar.

This conversion affects fewer than 2.5% of annual timesteps but imposes the structure of Gregorian calendar months onto a model with uniform 30-day months. While this ensures compatibility with the bias-adjustment approach used here, it may alter aspects of the model’s internal temporal structure. Alternative approaches, such as distributing the additional 5-6 interpolated days more evenly across the year using a continuous day-of-year mapping, could better preserve the intrinsic temporal characteristics of the 360-day calendar and represents a more appropriate approach for future implementations.

Users of the bias-adjusted HadGEM3 output should be aware that this model exhibits larger deviations from the reference variability compared to other HighResMIP experiments. Bias adjustment can reduce systematic mean biases but cannot fully compensate for structural deficiencies in simulated variability, and results should therefore be interpreted with appropriate caution.

All the datasets were interpolated to a 0.5° horizontal resolution. For variables such as tas, tasmin, tasmax, sfcWind, hurs, huss, ps, we used the bilinear remapping approach while for pr, prsn rsds and rlds a conservative remapping method was used following common practice in climate impact modeling^24,33^. The historical period was restricted to 1979 due to the limitation of the reference data. As stated in the method description of ISIMIP3BASD^39^, temperature extremes, tasmin and tasmax are first converted to temperature range (tasrange = tasmax - tasmin) and temperature skewness (tasskew = (tas - tasmin)/(tasmax - tasmin)). For solid precipitation (prsn), the ratio prsnratio = prsn/pr were also generated for bias adjustment.

### Bias Adjustment Methodology

The Inter-Sectoral Impact Model Intercomparison Project (ISIMIP) has developed and standardized its bias adjustments, with ISIMIP3BASD^39,40^ representing the most current employed by community^24,41^. ISIMIP3BASD^39,40^ employs a consistent bias-adjustment framework. For each grid cell and calibration window, a parametric distribution is fitted and evaluated using a Kolmogorov-Smirnov^42^ test. If the parametric fit is rejected, it automatically falls back to non-parametric empirical quantile mapping. This behaviour is an integral safeguard of the ISIMIP3BASD^39,40^ methodology ensuring the raw climate model projected changes remains physical consistency and preserved throughout the process^24,39^. The Trend preservation in ISIMIP3BASD^39,40^ operates at the quantile level: for temperature, the absolute difference (additive trend) between the historical and future period is preserved at each quantile of the distribution after bias adjustment. For precipitation, the ratio (multiplicative trend) between periods is preserved at each quantile. This means that the model-projected shift in the full distribution, including changes in the median, tails, and extremes are retained. ISIMIP3BASD^39,40^ preserves the relation within variables such as tas, tasmin, tasmax and also pr and prsn, by using an indirect adjustment approach.

For bounded variables such as relative humidity (hurs), which is physically constrained between 0–100%, ISIMIP3BASD^39,40^ prevents supersaturation by capping adjusted values at 100% and preserving the frequency of referenced saturation events (i.e., the proportion of time steps where humidity reaches 100%). Consequently, the variability of the bias-adjusted humidity is constrained, particularly over ocean areas where saturation occurs more frequently. This can reduce the ability of the bias adjustment to reproduce observed trends in relative humidity and should be considered a known limitation of the method when interpreting hurs results. The ISIMIP3BASD^39,40^ uses the best possible distributional approach depending on the variable’s characteristics making the methodology unique and valuable for consistency in adjusting multi variable and multi model experiments regardless of the climate type and location^24^. More information on the ISIMIP3BASD^39,40^ methodology can be found in Lange (2019, 2021).

However, ISIMIP3BASD^39,40^ does not preserve all statistical moments of a variable: for precipitation, preserving multiplicative quantile trends means that absolute changes in variance may differ from the raw model. ISIMIP3BASD^39,40^ uses a univariate approach in adjusting these biases at the grid cell level independently, this grid-by-grid approach may not fully preserve spatial coherence in climate fields, particularly for spatially-organized phenomena such as frontal systems, mesoscale convective complexes, or orographically-induced precipitation patterns^43,44^.

**Fig. 1:**
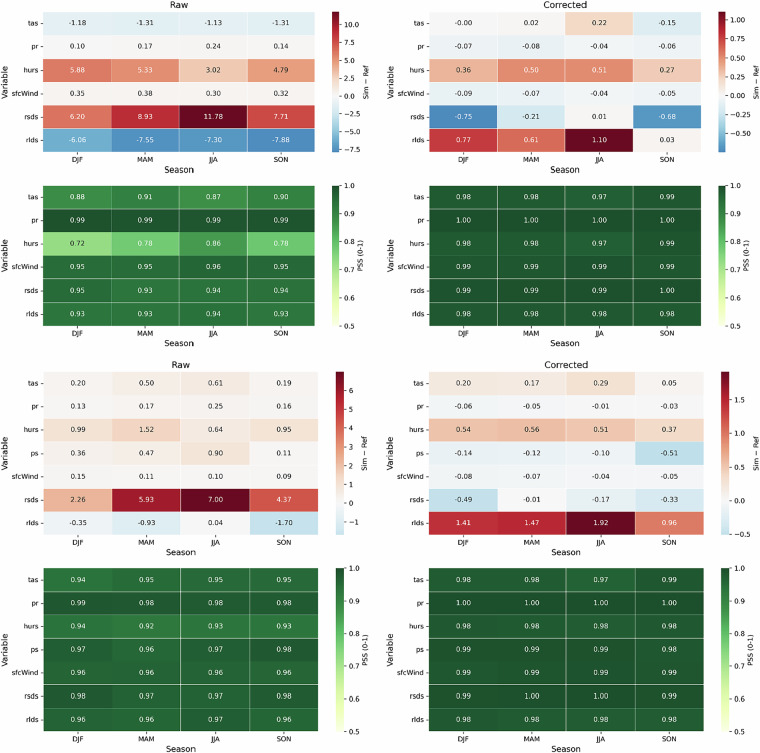
Split-Sample validation of the datasets for the period 2001–2014 based on calibration period 1979–2000. Upper plot for CNRM, bottom plot for EC-EARTH3P with difference between simulation and reference for both raw and corrected dataset as well as their Perkins Skill Score (PSS). Each panel pair shows global seasonal mean bias (Sim - Ref; top) and Perkins Skill Score (PSS; bottom) for both raw(left) and bias-adjusted (right) model output across six variables (tas, pr, hurs, ps, sfcWind, rsds, rlds) and four seasons (DJF, MAM, JJA, SON). Bias values represent the difference between model simulation and the W5E5 v2.0 reference dataset averaged over the validation period 2001–2014.

**Fig. 2:**
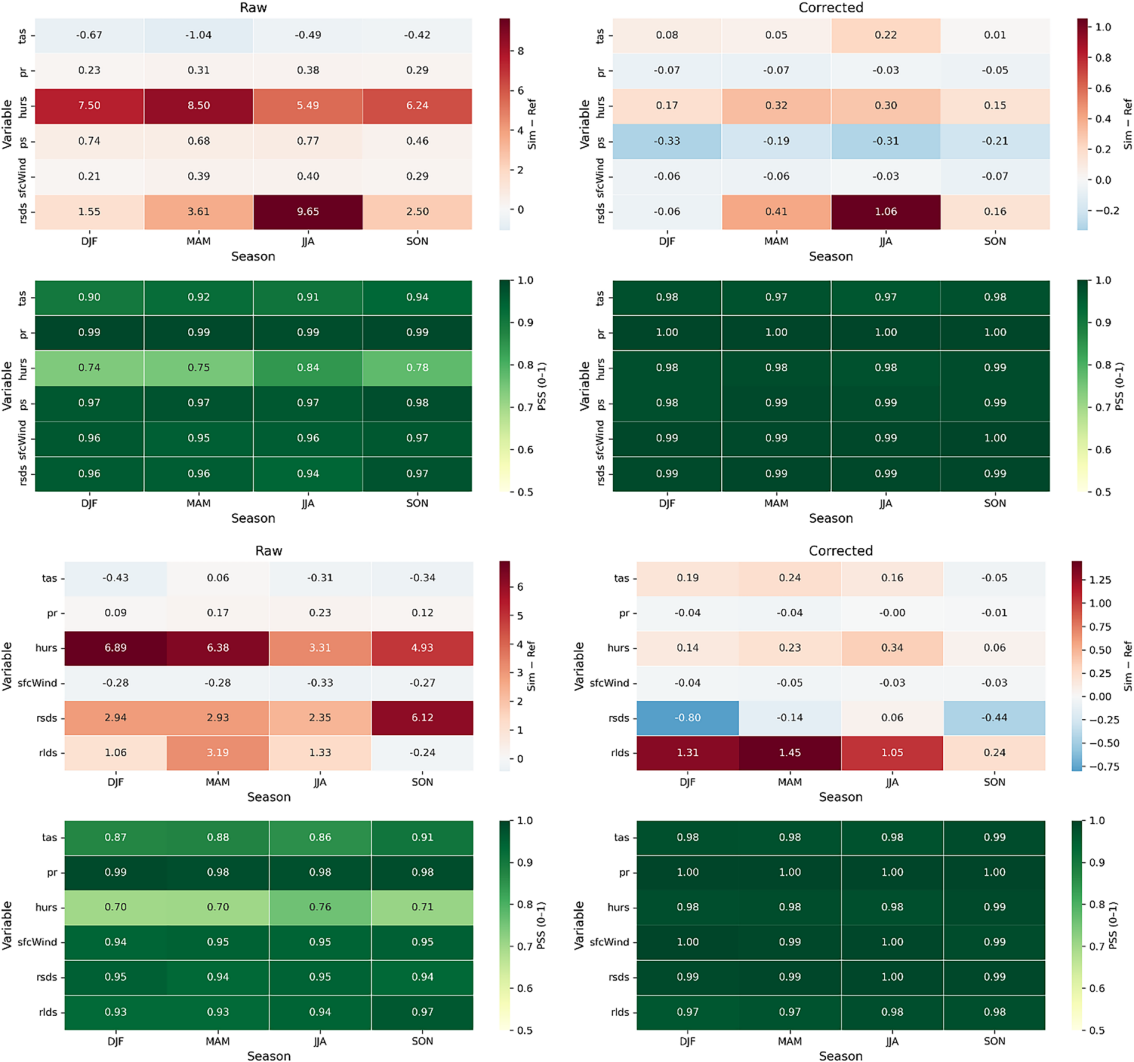
Same as Fig. 1, but for HadGEM3(top) and MPI-XR (bottom).

**Fig. 3:**
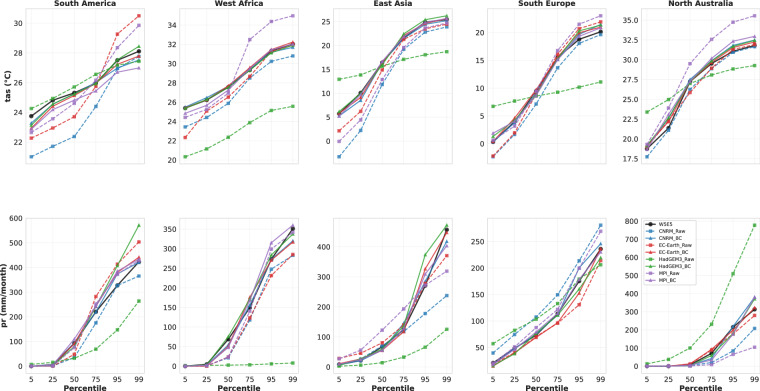
Monthly distributional plot showing the ability of the bias adjustment method to replicate that of the W5E5 v2.0 across all percentiles (5^th^, 25^th^, 50^th^, 75^th^ 95^th^, and 99th) for mean temperature (top) and precipitation (bottom) for five different climates across five global regions (South America, West Africa, East Asia, South Europe and North Australia). Validation period 2001–2014 based on 1979–2000 calibration. Note: Raw = unadjusted module simulations and BC = bias adjusted dataset.

A split sample validation test was conducted with 1979-2000 as training to adjust for 2001-2014 and validated against the reference for 2001–2014 (see Fig. 1, Fig. 2 and Fig. 3). For the bias adjustment, 1979-2014 (36 years) was then used as the training period for adjusting the historic period of the model simulations (1979-2014) and future projections (2015–2050)^38^.

### Postprocessing Datasets

After the bias adjustment, tasmin and tasmax were regenerated from the adjusted tas, tasrange and tasskew using the reverse of its equations. A similar approach is used to regenerate Snowfall (prsn) from bias adjusted pr and prsnratio. Specific humidity (huss) was not also bias adjusted directly using ISIMIP3BASD^39,40^, but was derived from bias adjusted Surface Pressure (ps), mean temperature (tas), and Relative Humidity (hurs) following the Arden Buck (1981)^45^ equations: saturation vapor pressure es = 6.1121 × exp[(18.678 - T/234.5)(T/(257.14 + T))] hPa, actual vapor pressure e = (hurs/100) × es, and specific humidity q = εe/(p - (1-ε)e), where ε = 0.622, T = tas in °C.

In ISIMIP3BASD^39,40^, defined variable-specific bounds are explicitly used to avoid physically implausible values after the bias adjustment. All the output variables were checked against these defined bounds as given in Lange (2019) and any out-of-bound value constrained. Instances of such physically implausible values after bias adjustment were negligible and in isolated model-scenario combinations (i.e., <0.00006% in sfcWind and rlds for HadGEM3 and MPI-XR).

We also selected 1° x 1° grid cells across five distinct climates representing diverse Köppen-Geiger climate zones^46^ to verify the performance of the bias adjustment in replicating same distribution as that of the W5E5 v2.0 dataset across these climate types. These selected regions include South America (14–15°S, 48–49°W), West Africa (10–11° N, 4–5° W), East Asia (30–31° N, 103–104° E), South Europe (44–45° N, 5–6° E), and North Australia (19–18° S, 137–138° E). These regions were chosen to span tropical (West Africa, North Australia), temperate (South Europe), continental (East Asia), and subtropical (South America) climates, ensuring validation across the major climate regimes where the dataset may be applied.

## Data Records

BC-HiRMIP^21^ datasets are deposited in the data management system at the Universität Hamburg and can be accessed using 10.25592/uhhfdm.18335. Across the four HighResMIP experiments, BC-HiRMIP^21^ includes up to 11 climate variables for the period 1979–2050. This is made up of ten variables each for the EC-Earth3P and HadGEM3 experiments and nine variables each for MPI-XR and CNRM experiments. In total, the dataset comprises of 76 individual files across two scenarios, with file sizes ranging from a minimum of ~2.4 Gb to a maximum of ~12 Gb depending on variable, and with a total combined size of ~721 Gb. A detailed technical description of the BC-HiRMIP^21^ dataset can be found in Table 2.

**Table 2:** Technical summary of variables included in the BC-HiRMIP dataset. Note: * indicates models missing ps and huss; ^+^ indicates models missing prsn.

Parameter	MPI-XR*	EC-Earth3P^+^	CNRM*	HadGEM3^+^
Variable Name (Unit)	tas (K), tasmax (K), tasmin (K), pr (kg m-2 s-1), prsn (kg m-2 s-1)^+^, hurs (%), huss (kgkg-1)*, rlds (Wm-2), rsds (Wm-2), sfcWind (ms-1), ps (Pa)*			
Long Name	Near-Surface Air Temperature, Minimum Near-Surface Air Temperature, Maximum Near-Surface Air Temperature, Precipitation, Snowfall Flux, Near-Surface Relative Humidity, Near-Surface Specific Humidity, Surface Downwelling Longwave Radiation, Surface Downwelling Shortwave Radiation, Near-Surface Wind Speed, Surface Air Pressure			
Hististorical & Highres-future	1979-2014 & 2015-2050			
Forcing Scenario	coupled future 2015-2050 using a scenario as close to CMIP5 RCP8.5 as possible within CMIP6			
Spatial & Temporal Resolution	0.5° × 0.5° (~50 km) Daily			
Spatial Coverage & Grid Type	180°W to 180°E, 90°S to 90°N Regular latitude-longitude			
File Format & Number of Files	NetCDF & 18	NetCDF & 20	NetCDF & 18	NetCDF & 20
Total Data Volume	~162 Gb	~199 Gb	~162 Gb	~199 Gb
Correction Method	ISIMIP3BASD v3.0.1			
Reference Dataset (Period)	W5E5 v2.0 (1979-2014)			

Each unique dataset is stored in a self-describing netCDF format, with a naming structure, *VariableAdjust_BHM-50_ESM_scenario_Variant-Label_W5E5_v2-1979-2014_day_yyyy-yyyy.nc*, where “VariableAdjust” represents the actual climate variables, e.g., prsnAdjust for prsn, rsdsAdjust for rsds, and tasmaxAdjust for tasmax. The expression “BHM-50” represents Bias-Corrected HighResMIP Dataset for Impact Assessment Studies (BC-HiRMIP^21^) and “ESM” stands for the Earth System model partaking in the HighResMIP experiment. The “Scenario” refers to historical or highres-future. The “*Variant-Label”* represents the simulation within the model’s ensemble used. The “W5E5_v2-1979-2014” for the reference dataset, and the training periods with “yyyy-yyyy” representing 1979-2014 for historical or 2015-2050 for highres-future. For example, prAdjust_BHM-50_MPI-ESM1-2-XR_hist-1950_r1i1p1f1_W5E5_v2-1979-2014_day_1979-2014.nc for pr and tasmaxAdjust_BHM-50_EC-Earth3P-HR_highres-future_r1i1p2f1_W5E5_v2-1979-2014_day_2015-2050.nc for tasmax.

## Technical Validation

### Validation

An independent validation was conducted on the bias adjustment using a split-sample test from the historical period. The split-sample validation (Figs. 1, 2 and 3) shows substantially improved bias across several variables in the evaluation period (2001–2014) relative to the raw model output. For temperature, bias-adjusted models show strong agreement with the reference across all percentiles and regions, with correlations consistently exceeding 0.95 and residual biases reduced to near-negligible levels for most models.

For precipitation, the bias adjustment performed well across the bulk of the distribution (5^th^-75th percentile), with bias-adjusted experiments closely tracking reference values. Performance degrades at the uppermost quantiles (95^th^-99th percentile), where divergence grows notably for HadGEM3-HM and MPI-XR. In the case of HadGEM3-HM, the raw model precipitation is noticeably poor across four out of the five regions, representing a fundamental baseline deficiency that renders reliable adjustment particularly challenging. More broadly, the degraded tail performance is consistent with known limitations of quantile-based bias adjustments. The limited training sample sizes with only 22 years available for calibration in the split-sample framework, l likely also introduced substantial sampling uncertainty into both the adjustment and the validation itself. For rlds, the performance of the bias adjustment was not consistent across all models. In particular, for EC-Earth, biases in some seasons increased slightly after correction, leading to slight overcorrection. As this behaviour is not observed consistently across all models, it likely reflects interactions between the bias-adjustment method and model-specific characteristics or internal variability, rather than a systematic limitation of the approach.

The split-sample validation, nonetheless, demonstrates that the bias adjustment reduces seasonal mean biases while preserving or slightly improving probabilistic skill in an independent period. This also showed that, the ISIMIP3BASD^39,40^ is temporally transferable since training on an earlier climate condition (1979-2000) still worked under a warmer, different circulation regime (2001-2014). This supports its application to future simulations, assuming approximate stationarity of model biases over the projection horizon.

### Spatial Evaluation

For evaluation, we focus on the 1985–2014 and 2021–2050 period (representing the final three decades of each scenario) to assess performance over a standard 30-year climatological period. We used spatial plots to evaluate the dataset against the reference W5E5 v2.0^27,33^, differences are calculated as raw simulations (or adjusted) minus the reference. The performance of the bias-adjustment across different variables, models and climate types are then evaluated. The spatial plots also give us the opportunity to evaluate the ability of the ISIMIP3BASD^39,40^ to preserve the raw climate model projected changes using the climate change signals. A robust bias adjustment method should be able to maintain the climate change projections of the raw climate model after adjustment. ISIMIP3BASD^39,40^ preserves climate change signals by applying quantile-level scaling factors derived from the historical period to the future period: additive scaling for temperature (preserving the absolute change at each quantile) and multiplicative scaling for precipitation (preserving the relative change at each quantile). This means the projected shift in the distribution, including changes in the mean, upper and lower tails, should be retained after bias adjustment. To verify this, we compared the raw model climate change signal (highres-future_raw_ minus historical_raw_) with the bias-adjusted climate change signal (highres-future_bc_ minus historical_bc_), confirming that quantile-level trends are not distorted after adjustment. At each grid cell, the climate change signal was defined as the difference between the climatological mean of the future period (2021–2050) and the historical period (1985–2014). This signal was computed separately for the raw model output and the bias-adjusted dataset. The raw climate change signal is given by ${\mathrm{Future}}_{\mathrm{raw}}-{\mathrm{Historical}}_{\mathrm{raw}}$, while the adjusted signal is ${\mathrm{Future}}_{\mathrm{bc}}-{\mathrm{Historical}}_{\mathrm{bc}}$. We then evaluated the difference between these two signals to assess whether the bias adjustment alters the model’s projected changes. A trend-preserving adjustment results in differences that are zero or close to zero, confirming that the original model trends are maintained. This preservation of trends is crucial for physically consistent climate change impact assessments^47^.

**Fig. 4:**
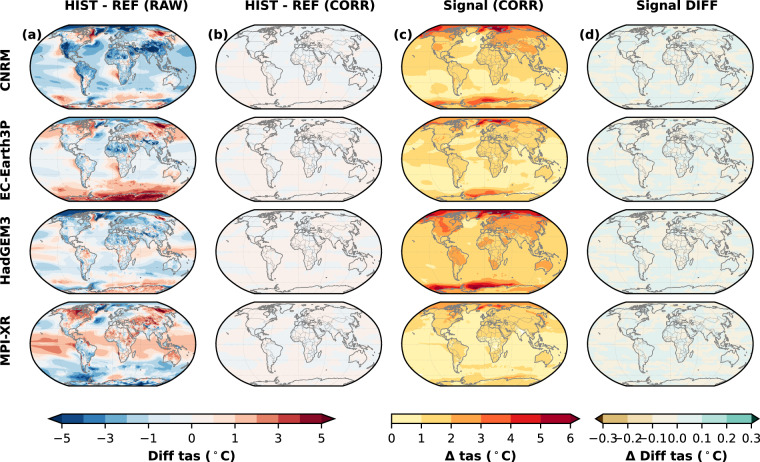
Spatial validation of mean near-surface air temperature (tas) bias adjustment performance for the four HighResMIP experiments (CNRM, EC-Earth3P, HadGEM3, MPI-XR, arranged top to bottom). Columns show: (**a**) differences in raw model simulations relative to W5E5 v2.0 (reference) for the evaluation period 1985-2014, (**b**) residual differences in bias-corrected datasets for the same period, (**c**) projected changes between future (2021–2050) and historical (1985–2014) periods in the corrected datasets, and (**d**) differences in projected change between raw climate model and bias-adjusted simulations, demonstrating trend preservation in the climate change signal.

**Fig. 5:**
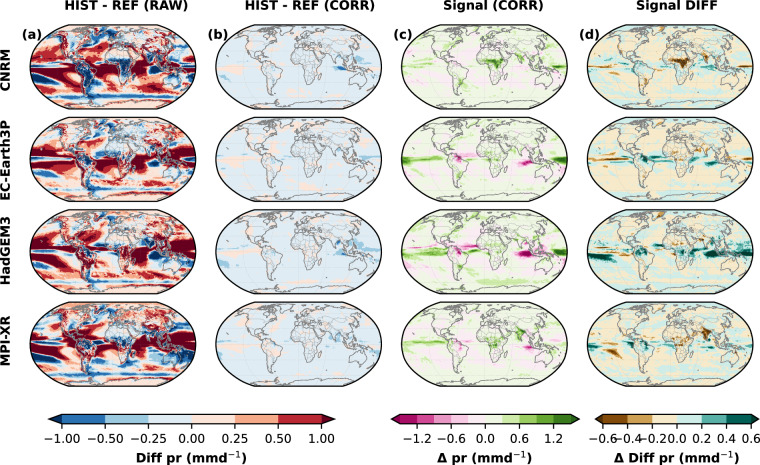
Same as in Fig. 4, but for pr.

**Fig. 6:**
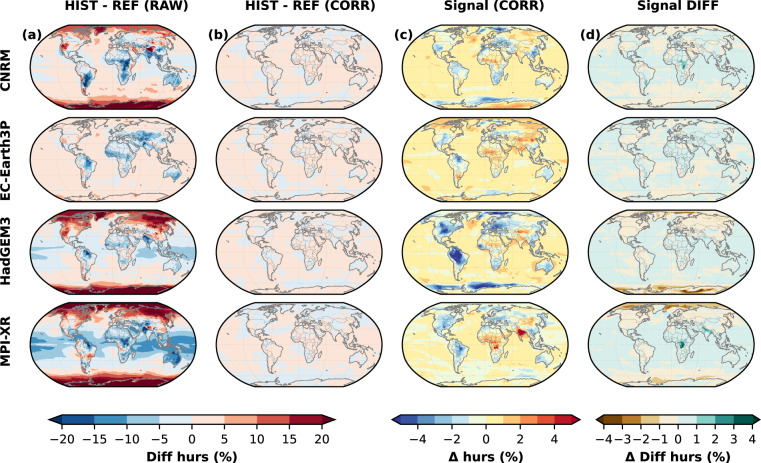
Same as in Fig. 4, but for hurs.

**Fig. 7:**
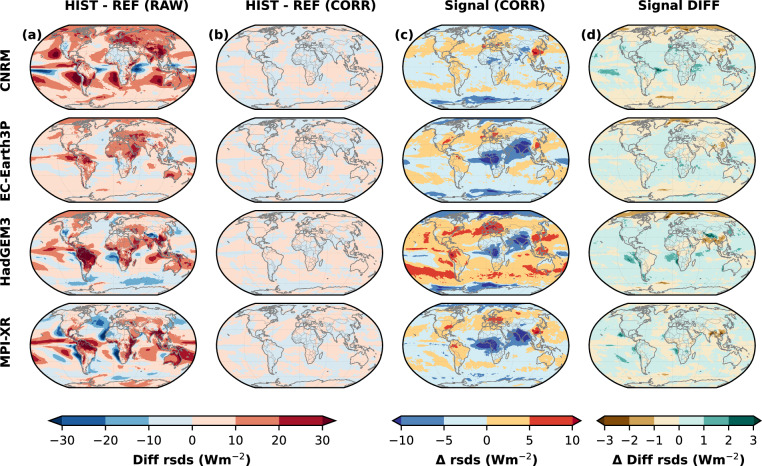
Same as in Fig. 4, but for rsds.

**Fig. 8:**
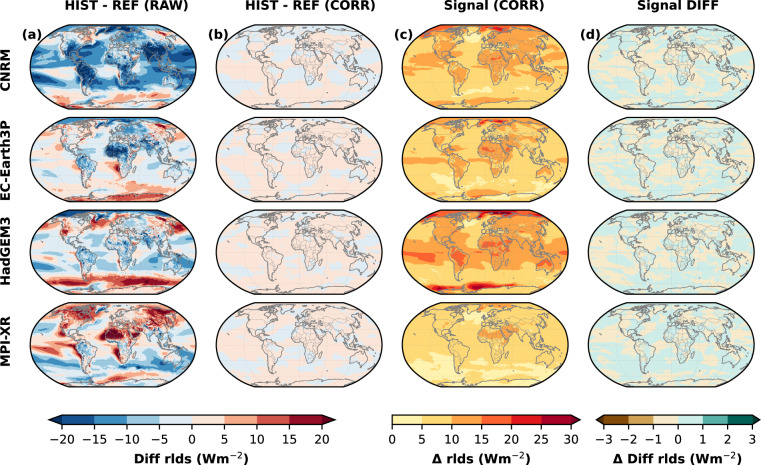
Same as in Fig. 4, but for rlds.

**Fig. 9:**
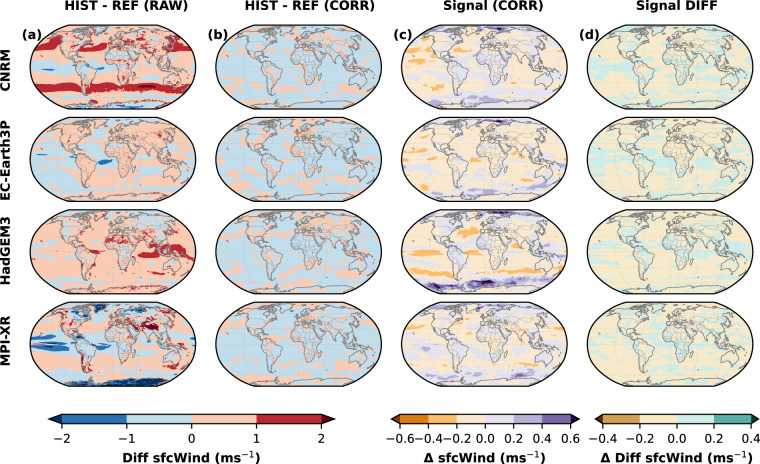
Same as in Fig. 4, but for sfcWind.

**Fig. 10:**
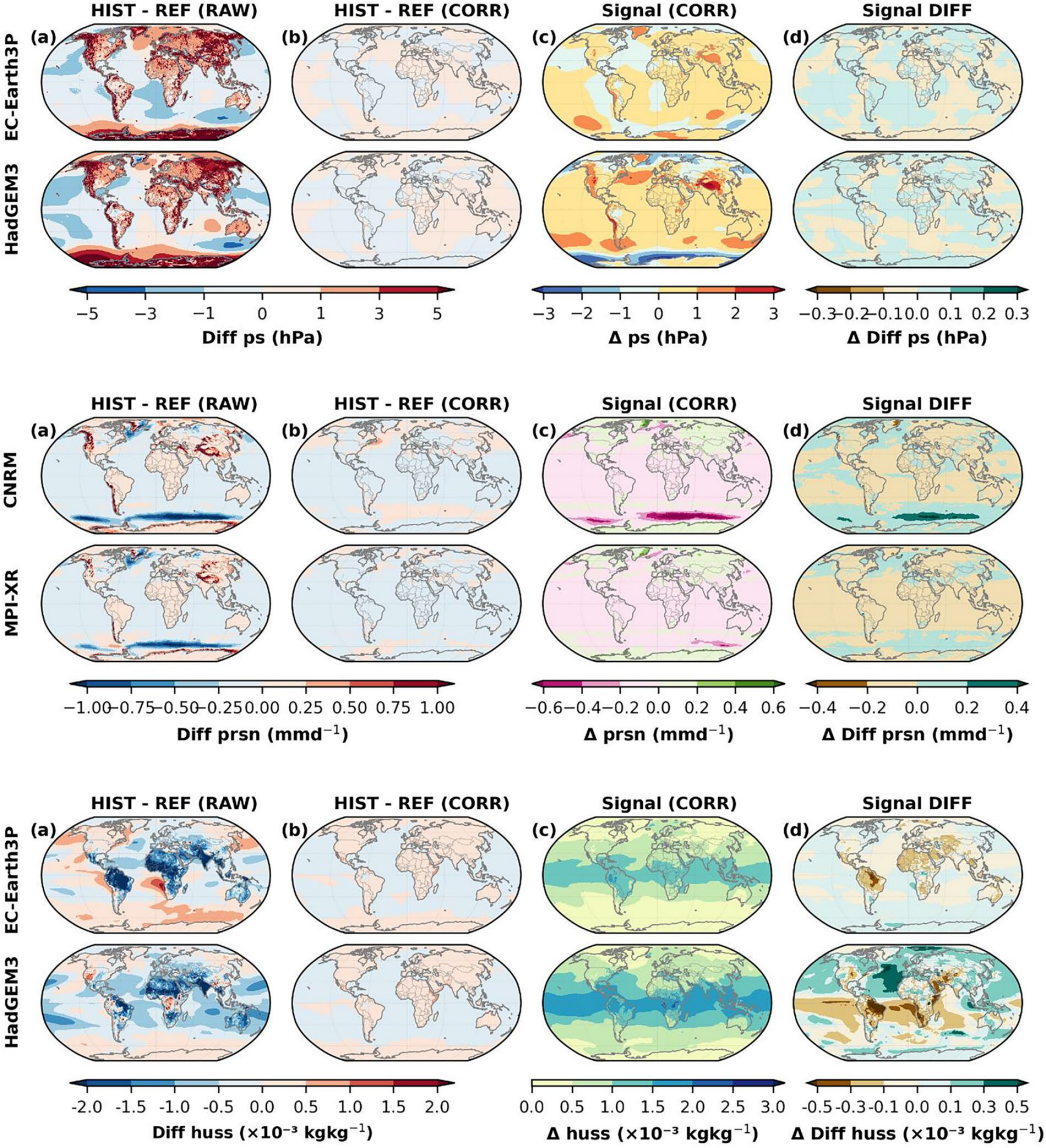
Same as in Fig. 4, but for ps, prsn and huss.

In addition to the spatial and distributional diagnostics shown in Figs. 4–10, we summarize key performance metrics for several of the variables globally for the four seasons, DJF, MAM, JJA, SON over the evaluation period 1985-2014 in Table S1. These metrics are the Correlation Coefficient (Corr): a measure of how well the temporal or spatial pattern of variations matches between the model and reference data. Bias and Percentage Bias (%_Bias): showing whether the models’ difference is systematically too high or too low and how large is the systematic error compared to the typical magnitude of the variable. Mean Absolute error (MAE): providing insight into how large a typical error is, regardless of direction or extremes. Root Mean Square Error (RMSE): showing whether there are occasional big mistakes that matter. Perkins Skill Score (PSS): this answers the question “do the two datasets (model and ref) represent the same overall climate/statistical behavior?”, the 95^th^ & 99^th^ Percentile Bias (B_p95/99) shows if the model systematically over- or underestimate the extremes, the Nash-Sutcliffe Efficiency (NSE) shows how well a model reproduces the time variability of the referenced data compared to using the referenced mean as a predictor, and Standard Deviation Ratio (Std_R) showing whether the model reproduces the spread (variability) of the referenced data.

For mean temperature (tas), as shown in Fig. 4, the raw HighResMIP experiments underestimated/overestimated at various locations. CNRM underestimated temperatures by up to -5 °C across the Arctic, Greenland, Himalaya, along 15°N −45°N and along the west coast of South America. EC-Earth3P over estimated temperatures up to + 5 °C across Antarctica and across the northeastern parts of Russia. HadGEM3 and MPI-XR had better agreement with the reference but was slightly colder over the Arctic ocean, overestimation across the oceans in the equatorial regions, temperatures above +3 °C in parts of Central Asia, northeastern Russia and over Canada. After bias adjustment, all these differences had been reduced to the barest minimum, with all experiments now showing negligible differences. For the climate change signal (Signal (CORR)), all four experiments show similar patterns of projected climate change over land but with slight differences at the poles and over the oceans. MPI-XR projects the oceans to warm by +1 °C, CRNM and EC-Earth3P by +1–2 °C and HadGEM3 by +2 °C. The climate change signal (Signal DIFF) in temperature, representing the absolute warming trend at each quantile of the distribution per grid cell, including the mean was preserved by the bias adjustment, as the difference between the raw and bias-adjusted signals (Signal Diff) for all experiments centred around zero across the full distribution.

In Fig. 5, precipitation (pr) in all four experiments show huge overestimations particularly over the ocean (between 30 °S and 30 °N), underestimation over northeastern parts of South America and parts of central Africa. These discrepancies are all reduced significantly after the bias adjustment. Precipitation projections over the continents appears between 0–0.6 mm/day, ~1.0 mm/day or higher over the oceans around the equator. The bias adjustment also significantly preserved the raw climate model projected changes (Signal Diff) over all areas except for isolated areas around the equator where some slight discrepancies still exist. Regions around the equator were slightly overestimated by between 0.2–0.6 mm/day depending on experiment, with HadGEM3 having the highest overestimations while CRNM the least. Slight reductions (−0.2 to −0.6 mm/day) also occurred mostly around isolated areas along the Inter-tropical Convergence zones (ITCZ) for all experiments, parts of west to central Africa, northeastern Greenland and over India for CRNM with MPI-XR also slightly underestimating over India. The slightly stronger signal over equatorial regions and the Indian subcontinent after bias adjustment could be related to precipitation differences being expressed as absolute values. In regions with high mean rainfall, absolute differences naturally appear larger, which can enhance the apparent magnitude of the adjustment in these areas. These discrepancies in the equatorial regions may also reflect model limitations related to the positioning of the ITCZ or other key circulation systems. In such cases, bias adjustments are unlikely to perform adequately or meaningfully address these underlying model deficiencies. Users should exercise caution when interpreting the dataset in this context.

Short and Long wave radiation (rsds and rlds) are also shown in Fig. 7 and Fig. 8. Overestimations of up to ±30 Wm^−2^ can be seen for rsds and up to ±20 Wm^−2^ for rlds, CRNM appears to be the experiment with the high under- or overestimation. The bias adjustment reduced these discrepancies to the barest minimum as shown in (b) of Fig. 7 and Fig. 8, where these differences become negligible. CNRM, MPI-XR and EC-Earth3P projects an increase in rlds of about +5–10 Wm^−2^ and up to +5 Wm^−2^ for rlds in the higher latitudes. HadGEM3 shows up about +15 Wm^−2^ for rlds and >+5 Wm^−2^ for rsds. All experiments project a reduction in rsds over central Africa, East to West coast of Asia down to about −5 Wm^−2^ or more. Signal differences remain close to zero over most regions, indicating that ISIMIP3BASD^39,40^ largely preserved the raw climate change signals for rlds and rsds, with localized deviations of up to ±2 W m^−2^. Similar performance pattern can be seen for Relative Humidity (hurs) (Fig. 6), Surface Wind (sfcWind) (Fig. 9), Snowfall (prsn), Surface Pressure (ps) and the derived Specific Humidity (huss) (all in Fig. 10).

## Temporal Evaluation

**Fig. 11:**
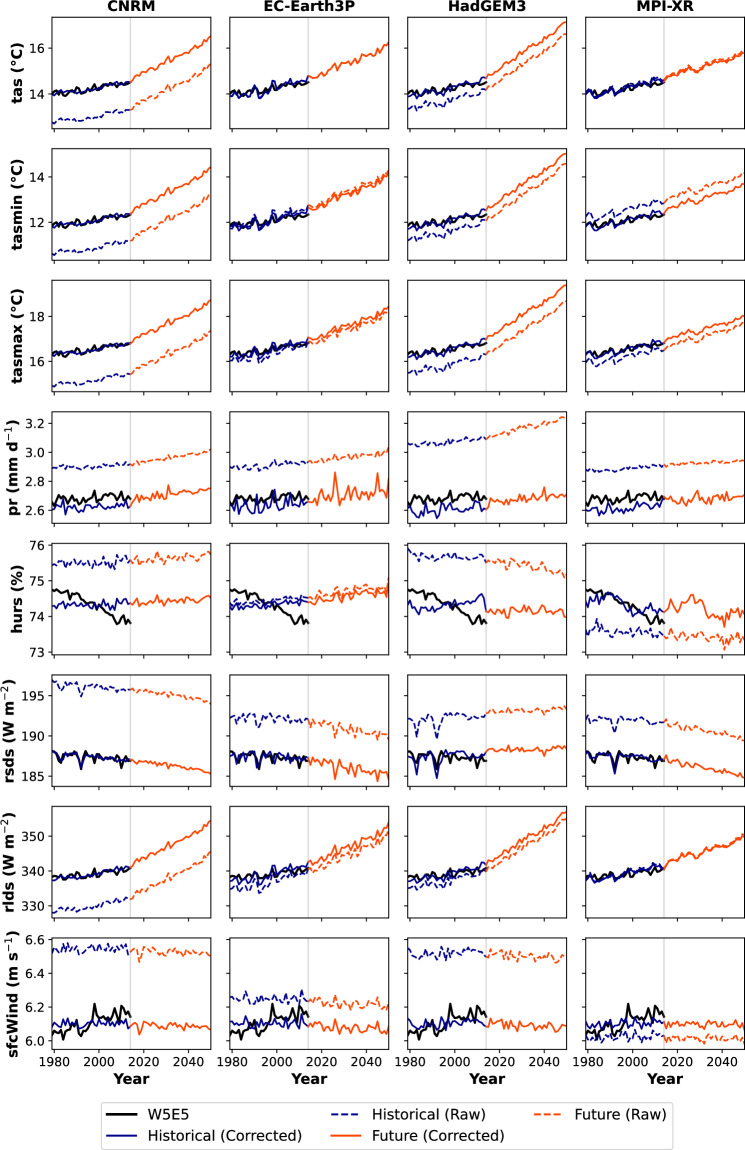
Global mean temporal distribution (including oceans) of tas, tasmin, tasmax, pr, hurs, rsds, rlds and sfcWind from 1979-2050 in descending order. HighResMIP experiments arrange from left to right: CRNM, EC-Earth3P, HadGEM3 and MPI-XR, each column representing variables various experiments. Note: The “raw” lines for tas in EC-Earth3P, rlds in MPI-XR coincides with the “corrected” lines.

**Fig. 12:**
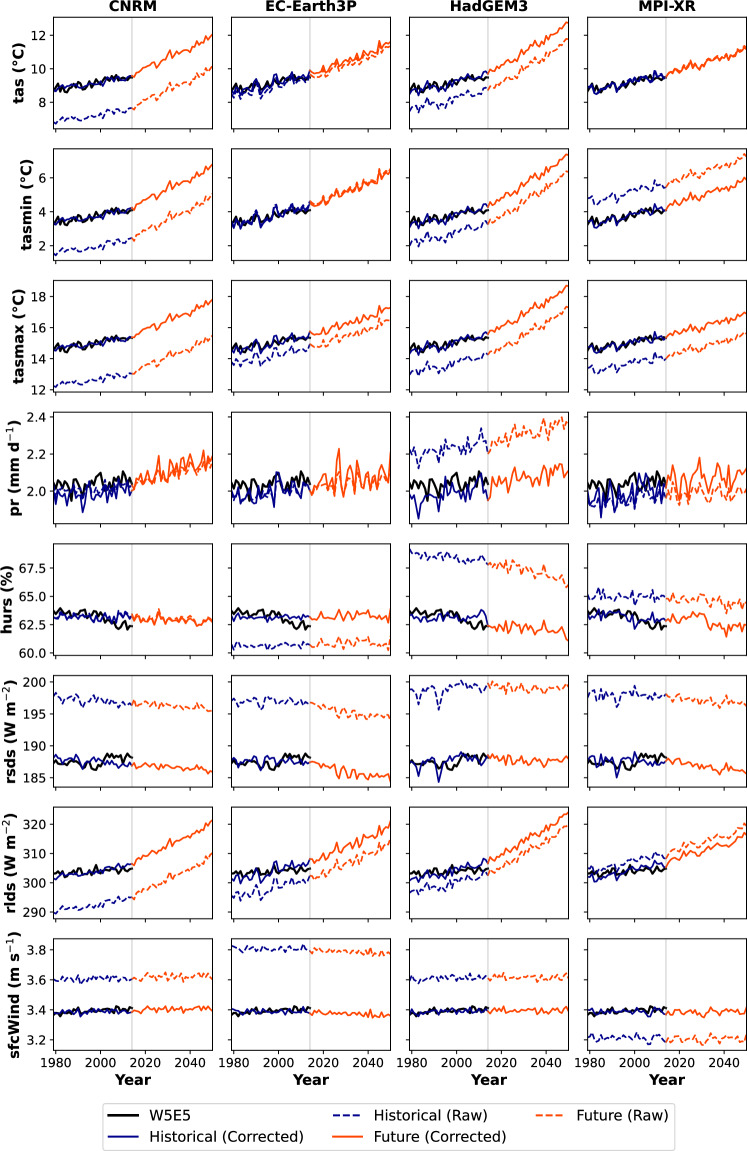
Same as Fig. 11, but only over land.

A before and after the adjustment global mean timeseries was computed (Fig. 11). We computed spatial averages using area weighting, so that each grid cell contributed according to its actual surface area. This was done by weighting each cell by the cosine of its latitude, which prevents high-latitude (polar) regions from being overrepresented. Figure 11 includes all global areas including all oceans. For temperature, the raw EC-Earth3P experiment performed better with tas, tasmin and tasmax showing the least difference with the reference over time. The CNRM experiment showed the most difference with temperatures being underestimated by ~-1 °C overall, HadGEM3 also underestimated for temperature while the MPI-XR slightly overestimated tasmin and also slightly overestimating tasmax. Precipitation was overestimated by up 0.3-0.5 mm/day for all experiments before bias adjustment. EC-Earth3P shows the least difference in simulated hurs, CNRM and HadGEM3 showed slight overestimated by up to ~+ 1%, while MPI-XR slightly underestimating by ~-1%. Overestimation of about ~+ 9 Wm^−2^ can be seen for CNRM, ~4 Wm^−2^ for EC-Earth3P, HadGEM3 and MPI-XR. Except for HadGEM3, all other models project a reduction in amount of rsds globally to about ~185 Wm^−2^ by mid-century. Underestimated by up to ~-10 Wm^−2^ in rlds can be seen for CNRM while the other three experiments showed very little differences. SfcWind was also overestimated by ~-5 ms^−1^ for CNRM and HadGEM3, up to ~+ 2 ms^−1^ for EC-Earth3P and underestimating for ~-1ms^−1^ for MPI-XR. The bias adjustment reduced all these with significant accuracy across different variables. A separate figure (Fig. 12, land-only), shows slightly different dynamics when the ocean contributions to these differences are ignored.

**Fig. 13:**
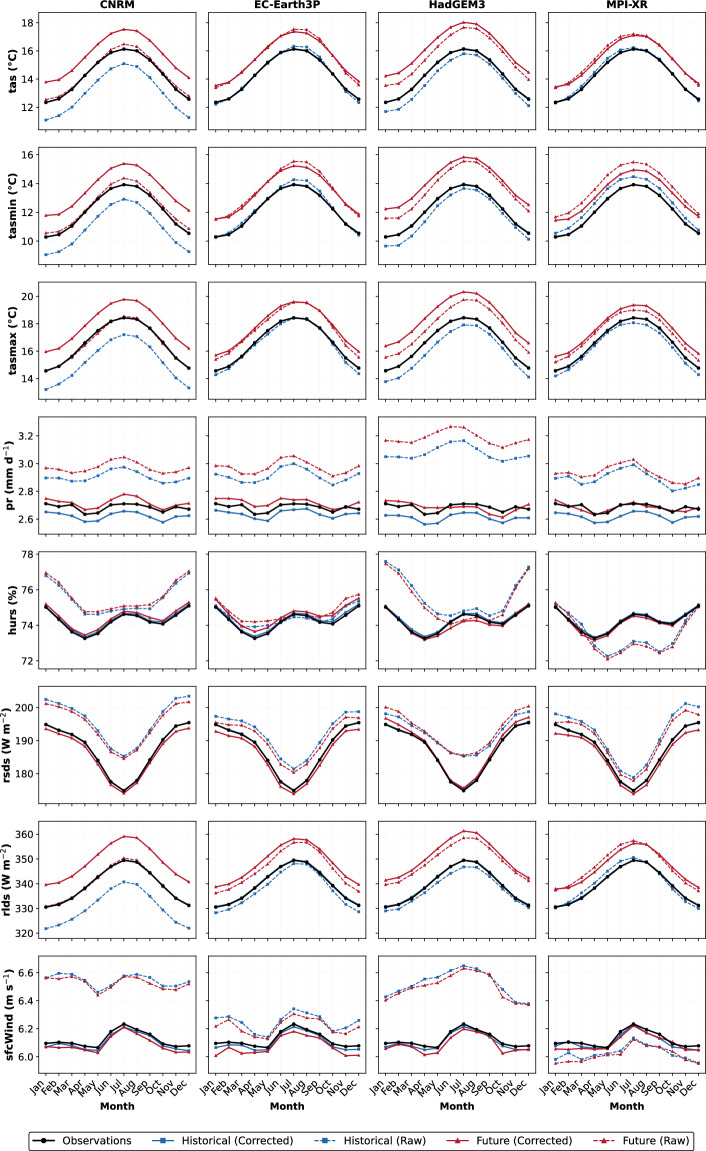
Monthly Climatology of tas, tasmin, tasmax, pr, hurs, rsds, rlds and sfcWind from 1985-2014 (historical) and 2021-2050 (highres-future) in descending order.

While all GCMs successfully captured the seasonal phases of the observed variables well, significant systematic biases exist in the raw outputs as demonstrated in the monthly climatological distribution (Fig. 13). These discrepancies were effectively minimized across all variables and experiments using the ISIMIP3BASD^39,40^. For precipitation (pr), all experiments exhibited a systematic wet bias (~0.2–0.3 mm/day), which was largely eliminated post-adjustment, with only a negligible residual underestimation of ~0.05 mm/day. Surface Wind Speed (sfcWind) showed varying levels of model drift; CNRM and HadGEM3 required significant downward adjustments from ~6.5 to 6.1 m/s, whereas MPI-XR showed higher initial fidelity. Notably, the bias adjustment adjusted temperatures (tas, tasmin, tasmax) and longwave radiation (rlds) upward while preserving seasonal patterns. Short wave radiation (rsds) saw a drop of ~5 W/m^2^ indicating an overestimation by all the GCMs.

### Evaluation Insights

**Fig. 14:**
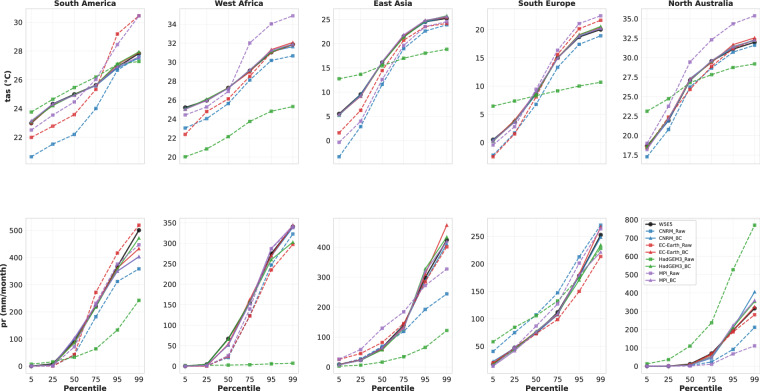
Same as in Fig. 3, but for the period 1985-2014.

**Fig. 15:**
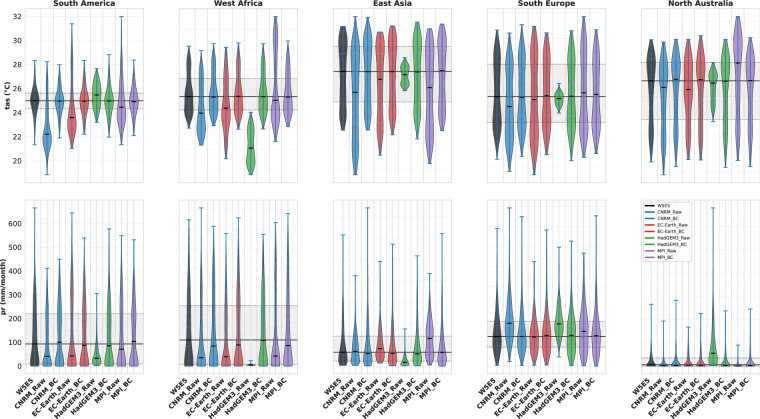
Same as Fig. 3, but using violin plots to show the actual distribution and shape of the dataset before and after bias adjustment against the W5E5 v2.0 dataset. Note: Gray area shows the 25^th^, 50^th^ and 75^th^ percentile of the reference distribution.

The impact assessment community often requires biases to be removed across the distributions and therefore prefers quantile mapping approaches over simpler methods dealing with single moments of the distributions. For this, we evaluate our dataset to see if they represent well the distribution of the reference data across all percentiles making it very vital for use in the impact assessment community. For each climate zones selected, area-weighted regional mean time series were derived from the reference, raw simulations, and the bias-adjusted dataset. Empirical percentiles (5^th^–99^th^) of these time series were then compared to assess how well the experiments reproduce the referenced distribution and its extremes as shown in Fig. 14 and Fig. 15.

Mean temperature (adjusted) distribution across all percentiles (Fig. 14) were simulated with remarkably high accuracy regardless of the region or climate type. The raw simulations distributions fail in matching that of the referenced, especially when assessing climate extremes where a mismatch for the tails of the distributions becomes noticeably clear. Some of the raw simulations such as the HadGEM3 experiment captured only few of these percentiles and failed to adequately represent the rest well. HadGEM3 shows substantially larger discrepancies in simulated temperature/precipitation variability relative to W5E5, compared to the other HighResMIP experiments here (Fig. 14 and Fig. 15). This is particularly evident across several regions, where the model’s variability structure deviates outside the range of the remaining ensemble. While bias adjustment partially reduces systematic distributional errors, it does not correct for fundamentally misrepresented variability^47^, and the bias-adjusted HadGEM3 output should therefore not be treated as equivalent in quality to the other adjusted HighResMIP experiments for applications sensitive to temperature/precipitation variability.

The adjusted precipitation reproduced well the referenced distributions, including large parts of the extremes across West Africa, East Asia, and Southern Europe, although some notable discrepancies still remain in some of these regions for the rare extremes. For South America and Australia, BC-HiRMIP^21^ shows high accuracy up to the 95^th^ percentile, but for the rare extreme events (>95^th^ percentile), some clear underestimations of events can be seen, though magnitudes varies depending on experiment (Fig. 14). The bias-adjusted precipitation shows degraded performance at the uppermost quantiles (above the 95th percentile) for some areas, which is consistent with known limitations of quantile-based bias adjustments in representing distributional tails. This behaviour has been documented in several studies, Themeßl *et al*. (2012) showed that empirical-statistical correction methods perform well across the bulk of the distribution but show increasing deviations toward the extremes due to limited sample sizes in the far tails^48^, while Switanek *et al*. (2017) also noted that standard quantile mapping can distort heavily skewed distributions such as precipitation at high quantiles^49^. Berg *et al*. (2024) further argued that extreme quantiles cannot always be reliably corrected and should be accompanied by explicit uncertainty bounds^50^. The deviations observed in BC-HiRMIP^21^ therefore reflect an inherent limitation of empirical bias-adjustment approaches rather than a general methodological failure, and users should exercise caution when interpreting results in these distribution tails or analyses specifically targeting rare precipitation extremes without additional uncertainty assessment^43^.

The violin plot (Fig. 15), shows the distributional shape of the referenced, raw simulations and the adjusted dataset, providing a comprehensive visual assessment of probability density functions before and after bias adjustment^51^. The bias adjustment transformed the raw simulations with huge distributional mismatches to mirror the referenced dataset. For mean temperature, an example of the mismatch between HadGEM3 simulations and the referenced before and after the bias adjustment can be seen, ranging from adjustment of underestimations to entire distributional spread of the data after. Similar improvements can be seen in the precipitation, e.g. HadGEM3 failed to capture distributions or shape accurately over West Africa, where a large transformation could be seen in the same model’s distribution after being bias adjusted.

Despite these improvements, BC-HiRMIP^21^ is still based on a univariate bias-adjustment framework. ISIMIP3BASD^39,40^ preserves selected inter-variable relationships (e.g., between tas, tasmin, tasmax and between pr and prsn), it does not explicitly constrain the full multivariate dependence structure across all variables and grid cells^43,44^. This limitation is particularly relevant for compound extreme analyses, where temporal co-occurrence and intensity relationships between variables (e.g., concurrent heat and drought, or precipitation and wind extremes) may not be fully preserved^52,53^. Users investigating multivariate extremes or compound hazards, or other applications highly sensitive to spatial patterns (e.g., river basin hydrology, regional agricultural assessments), should evaluate whether spatial structures are adequately represented for their specific domain before use. Using W5E5 v2.0^33^ as reference means BC-HiRMIP^21^ inherits all the uncertainties associated in this model output, which could occur particularly in data-sparse regions such as high-latitude areas, complex terrain, and parts of Africa and South America where gauge networks are limited and modellers rely on huge parametrizations^54^. These uncertainties propagate through the bias adjustment and may affect the reliability of bias-adjusted projections in regions with sparse observational coverage^55^. Users are advised to exercise caution when applying BC-HiRMIP^21^ in such regions and should consider validation against independent regional datasets where available. Spuler *et al*. (2023) showed that even trend-preserving bias-adjustment methods such as ISIMIP3BASD^39,40^ can substantially modify dry extremes at some locations, particularly in arid regions^41^. These changes can be locally large (50-100%) and arise from a structural mismatch between the empirical CDF-based trend preservation and parametric quantile mapping used for the bias adjustment^41^. Parametric distributions (e.g. gamma for precipitation) may inadequately represent tail behaviours, leading to amplified distortions in low-precipitation extremes despite preserved mean trends^41^. Users of BC-HiRMIP^21^ are nonetheless advice to take this into account before using the dataset for their own research.

BC-HiRMIP^21^ provides a robust, globally bias adjusted dataset covering 11 climate variables from across four HighResMIP experiments for historical and highres-future scenarios with a wide range of climate sensitivities. Validations ranging from spatial coverages across diverse climate types and locations, trend-preserving ability of the methodology used to its capability of simulating similar percentiles and shape of the referenced dataset. BC-HiRMIP^21^ brings to the impact assessment and the entire climate research communities a base dataset, that provides suite of variables for health, hydrology, agriculture, energy, ecosystems and climate extreme research. The data’s multi-variate, multi-model coverage together with its’ use of same reference and bias adjustment methodology contributes to reducing uncertainties that usually arises from running simulations with inconsistent datasets. BC-HiRMIP^21^ is the first comprehensive global bias adjusted climate dataset based on the HighResMIP experiments at 0.5° resolution and without substantial statistical downscaling, thereby offering the climate research and services community an alternative to quantify uncertainties plaguing the current datasets.

## Supplementary information

Supplementary Table S1

## Data Availability

The BC-HiRMIP dataset is deposited in the data management system at the Universität Hamburg and can be accessed using 10.25592/uhhfdm.18335.

## Code availability

The regridding of datasets to the reference grid and other computations were performed using Climate Data Operator (CDO, version 2.4.1) from the Max Planck Institute of Meteorology, it can be accessed using (https://code.mpimet.mpg.de/projects/cdo, Last access: 8 December 2025). The ISIMIP3BASD v3.0.1 script is publicly available and can be obtained from the repositories (https://zenodo.org/records/7151476). The study uses the Python implementation of the ISIMIP3BASD from the ibicus package, which can be accessed at (https://github.com/ecmwf-projects/ibicus, Last access: 8 December 2025). The scripts used for the bias adjustment, and in deriving Relative Humidity (huss) is available and can be accessed at (10.25592/uhhfdm.18335).
